# Prevalence, knowledge and attitude of prohibited substances use (doping) among Saudi sport players

**DOI:** 10.1186/s13011-016-0058-1

**Published:** 2016-04-16

**Authors:** M. Al Ghobain, M. S. Konbaz, A. Almassad, A. Alsultan, M. Al Shubaili, O. AlShabanh

**Affiliations:** Department of Medicine, College of Medicine, King Saud bin Abdulaziz University for Health Sciences, PO Box 90068, Riyadh, 11321 Saudi Arabia; Saudi Arabian Anti-Doping Committee, Riyadh, Saudi Arabia; Saudi Food and Drug Authority, Riyadh, Saudi Arabia; Department of Medicine, South Armed Forces Hospital, Abha, Saudi Arabia; Departments of Pharmacology and Toxicology, College of Pharmacy, King Saud University, Riyadh, Saudi Arabia

## Abstract

**Background:**

To estimate the lifetime prevalence and address the attitudes and knowledge of using prohibited substances (doping) among sport players in Saudi Arabia.

**Methods:**

A cross-sectional survey carried out using systematic random sampling technique among Saudi players of variable sports attending the sport clubs, stadiums, and sport fields (70 sport clubs, 22 types of sports belong to 22 Saudi sport federations were visted in 18 cities from all regions of Saudi Arabia).

**Results:**

A total of 1142 male sport players were interviewed with main age of 24. The prevalence of using prohibited substances (doping) was 4.3 %. The main reason for using prohibited substances was to improve performance (69 %). The prevalence of using food supplements (not prohibited) was 38.4 %. Among the players, 30 % of them believe that such behavior is against the spirit of sport while 70 % of the players are aware of punishment against doping. 65 % of players admitted that they received advice on prohibited substances. Higher rate of using prohibited substances (doping) among Saudi players was associated with low education, age below 20 years, previous use of food supplements and lack of punishment awareness.

**Conclusion:**

Using prohibited substances (doping) among Saudi sport players is common. Players believe that such use is against the spirit of the sport and they are aware about its punishment, despite this, they are still using prohibited substances.

## Background

The use of prohibited (banned) substances (doping) is considered to be a major global problem among sports players in and out of sports events. For several decades, many professional athletes were using prohibited substances especially during competitions for performance enhancing purposes. The use of these prohibited substances can lead to serious and harmful health hazards, including anti-social behavior, dependencies and even deaths [1]. Several cases died as consequences of such use. For example, in 1967, the post mortem analysis of Tom Simpson–a professional British cyclist- found that he had taken amphetamines and alcohol combination [1]. Some athletes are aware of the substances’ health hazards at variable degrees; however, others need more awareness and education on numerous prohibited substances [2].

In 1999, World Anti-Doping Agency (WADA) was established to regulate, monitor and control substance use around the world. The WADA produces a prohibited list of banned substances that is updated annually and documents the prohibited substances and methods of use inside and outside competition. Currently, 2 of the following 3 criteria must be met for a substance to be included on the prohibited list: (1) the substance increases or has the potential to increase performance; (2) the substance represents an actual or potential health risk to the athlete; and (3) the substance violates the spirit of sport [3]. In 2004, the Saudi Arabia Anti-Doping Committee (SAADC) was established to act as the independent national Anti-Doping organization for Saudi Arabia. The SAADC works in planning, coordinating, implementing, monitoring, and advocating improvements in doping control in Saudi Arabia [4].

There is insufficient data in the Middle East including Saudi Arabia regarding prevalence, attitude, and knowledge of athletes about prohibited substances use, types of substances most commonly used, reasons for use, athletes who are most at risk to use, and types of sports associated with use. Hence, there is a major need to conduct a study in Saudi Arabia to fill the mentioned gaps in literature and to put future recommendations that would address these issues. The purpose of the present study is primarily to estimate the lifetime prevalence of prohibited substances use among sports players in Saudi Arabia and to investigate the attitude and knowledge of Saudi Arabian sport players about prohibited substance use.

## Methods

A cross-sectional survey using a systematic random sample among all Saudi male sport players above the age of 15 years presenting to sports clubs, stadiums, sports fields and playgrounds affiliated with Saudi Sport Federations and the General Presidency for Youth Welfare in the Kingdom of Saudi Arabia. The study was carried out by distributing the constructed survey among 1200 Saudi players of 22 variable types of sports attending 70 different sport clubs, stadiums, sports fields and playgrounds in 18 cities from all regions of Saudi Arabia throughout the season of 2015. From each particular sport type, we selected players in random sample selection technique represented a proportion to the sport players of each sport type to the total number of the registered players of all types of sport e.g. 390 football players were selected randomly out of 16,779 registred football players (34.2 %).

After a review of the literature, a questionnaire was self-constructed by the authors. The questionnaire intended to investigate the participants’ attitude and knowledge towards using of prohibited substances, examine their knowledge and awareness about the punishment attributable to use, and estimate the prevalence of use. The questionnaire was reviewed and validated by experts from the members of SAADC. It was then pilot tested to ensure and determine clarity. Eligible participants were approached and consent was taken after explaining the aims and objectives of the study. Participants’ names were not recorded and data was kept confidential to protect their privacy. Data was not used for purposes other than the objectives of the study. The study protocol received ethical approval by the Ethical Research Committee (IRB) of King Abdullah International Medical Research Center at King Saud bin Abdulaziz University for Health Sciences, Riyadh, Saudi Arabia.

### Statistical analysis

Demographic variables were summarized and reported across the study cohort using descriptive statistics. Continuous variables were summarized and reported in terms of means and standard deviations. Categorical variables were summarized and reported in terms of frequency distribution. Since the outcome of interest was dichotomous “Ever used any type of prohibited substances” simple binary logistic regressions were then conducted to study the association between age, education, division of league, reasons for use, use of supplements, behavior and awareness of punishment with the use of prohibited substances. For that purpose unadjusted bivariate analyses were conducted independently for all exposures to identify the presence of an association with the use of prohibited substances. Chi-square tests were used for categorical variables. Associations between exposures and outcome were considered statistically significant for *P* ≤ 0.05. Analyses were conducted using SPSS (V.22).

## Results

Table 1 shows the total, frequency and percentage of sports players by type of sports. The total number of sports players invited were 1200 players, in whom, 1142 players accepted to be interviewed (response rate of 95 %) out of the total 49,074 professional players registered in the Saudi Sport Federations and the General Presidency for Youth Welfare in Saudi Arabia (ministry of the sport).

**Table 1:** Frequency and percentage of sports players by type of sports

Sports type	Number of registered players	Number of interviewed players	Percentage of the players
Football (soccer)	16,779	390	34.2 %
Volleyball	5319	121	10.6 %
Athletics (track & field)	4437	103	9 %
Table Tennis	3474	80	7 %
Handball	3198	75	6.5 %
Basketball	2450	57	5 %
Karate	2325	55	4.8 %
Swimming	1903	45	3.9 %
Tennis	1870	44	3.8 %
Taekwondo	1509	35	3.1 %
Judo	1033	24	2.1 %
Gymnastics	627	15	1.3 %
Cycle	623	15	1.3 %
Wrestling	603	14	1.2 %
Weight lifting	579	13	1.1 %
Water polo	491	11	1 %
Boxing	490	11	1 %
Duel	446	11	0.9 %
Bodybuilding	428	11	0.9 %
Special needs	400	10	0.8 %
Archery	50	1	0.1 %
Shooting	40	1	0.08 %
	49,074	1142	100 %

Our results showed that the most common type of sports among Saudi players was football 16,779 (390, 34.2 %) followed by volleyball 5319 (121, 10.6 %) then Athletics 4437 (103, 9 %).

All interviewed players were males with a mean age of 24, sd = 0.19 (range from 15 to 45 years). Of them, 54.7 % were players of premier division league and 29.6 % had a university educational level. Among all interviewed players, 38.4 % of the them admitted using any type of food supplement with the most common type being vitamin pills (62 %) followed by protein powders (28.7 %) then minerals (9.3 %).

The lifetime prevalence of prohibited substances use (doping) among sports players in Saudi Arabia is 4.3 %. The majority (65.6 %) were advised against prohibited substances mostly by club physiotherapist (32.5 %) followed by club trainers (30.8 %). Among all interviewed players, 35 % have been tested for the use of prohibited substances, 5 % are willing to use any prohibited substances in the future and 22 % know other players in sport who using prohibited substances.

The most common reason stated for using prohibited substances was to increase performance (69.4 %) followed by social recognition (17 %). Almost one-third of the players think that using prohibited substances is considered dangerous to health (36.7 %), against the spirit of sport (33.1 %) and dishonest (30.2 %). 77.5 % were aware of the punishment attributable to prohibited substances use and 67.6 % thought that the punishment is fair (Table 2). Use of prohibited substances is significantly associated with below university education level (X ^2^ = 15, df =1, *p*-value <0.0001), age less than 20 years (X ^2^ = 15, df =1, *p*-value <0.0001), the previous use of any type of supplements (*p*-value <0.0001) and lack of punishment awareness (X ^2^ = 15, df =1, *p*-value <0.0001) (Table 3). Though risk differences were really quite small on the order of 1 or 2 percentage points but they were significant.

**Table 2:** Prevalence, knowledge and attitude of prohibited substance use among Saudi sports players

Variable	Category	*N* (%)
Age (Mean ± SD)		24.23 ± 0.19
League	Premier division	(625) 54.7 %
	Division one	(372) 32.5 %
	Division two	(88) 7.7 %
	Others	(57) 5 %
Educational level	Illiterate	(2) 0.2 %
	Elementary	(22) 1.9 %
	Intermediate	(156) 13.7 %
	Secondary	(624) 54.6 %
	University	(338) 29.6 %
Ever used any type of food supplement	Yes	(439) 38.4 %
	No	(703) 61.6 %
Type of food supplement	Vitamin pills	(302) 62 %
	Minerals	(45) 9.3 %
	Protein powders	(140) 28.7 %
Ever used any type of prohibited substances	Yes	(50) 4.38 %
	No	(1092) 95.62 %
Advice on prohibited substances	Yes	(750) 65.6 %
	No	(392) 34.4 %
Source of advice	Club physiotherapist	(244) 32.5 %
	Fitness trainer	(231) 30.8 %
	Media	(87) 11.65 %
	Friends	(145) 19.41 %
	Other	(43) 5.73 %
Ever tested for prohibited substances	Yes	(406) 35.6 %
	No	(736) 64.4 %
Times being tested	Once	(223) 54.93 %
	Twice	(110) 27.09 %
	Three times	(73) 17.98 %
Willing to use prohibited substances in the future	Yes	(58) 5.1 %
	No	(1084) 94.9 %
Know of players using prohibited substances	Yes	(253) 22.2 %
	No	(889) 77.8 %
Reason for using prohibited substances	Increase self-confidence	(92) 8.1 %
	Social recognition	(194) 17 %
	Protect health	(64) 5.6 %
	Improve performance	(792) 69.4 %
Behavior of using prohibited substances	Dishonesty and cheating	(345) 30.2 %
	Dangerous to health	(419) 36.7 %
	Against the spirit of sport	(378) 33.1 %
Supplements help being successful in sports	Agree	(485) 42.5 %
	Disagree	(657) 57.5 %
Use could violate regulations	Agree	(578) 50.6 %
	Disagree	(564) 49.4 %
Use constitutes cheating	Agree	(567) 49.6 %
	Disagree	(575) 50.4 %
Punishment awareness	Yes	(885) 77.5 %
	No	(257) 22.5 %
Punishment rate	Weak	(123) 13.9 %
	Fair	(598) 67.6 %
	Strong	(164) 18.5 %

**Table 3:** Binary logistic regression of characteristics with use of prohibited substances

Demographic characteristic	Category	Use of prohibited substances (*N*) %	*P* value
Educational Level	University	(11) 3.3 %	<0.0001*
	Below university	(39) 4.8 %	
Age	<20 years	(17) 4.8 %	<0.0001*
	20–30 years	(27) 4.7 %	
	>30 years	(6) 2.8 %	
Reasons for use prohibited substances	Increase self-confidence	(5) 5.4 %	0.128
	Social recognition	(14) 7.2 %	
	Protect health	(3) 4.7 %	
	Improve performance	(28) 3.5 %	
League	Premier division	(29) 4.6 %	0.766
	Division one	(16) 4.3 %	
	Division two	(2) 2.2 %	
	Others	(3) 5.2 %	
Ever used any type of supplement	Yes	(32) 7.3 %	<0.0001*
	No	(18) 2.5 %	
Advice on prohibited substances	Yes	(33) 4.4 %	0.99
	No	(17) 4.3 %	
Behavior of using prohibited substances	Dishonesty and cheating	(12) 3.5 %	<0.0001*
	Dangerous to health	(17) 4 %	
	Against the spirit of sport	(21) 5.5 %	
Punishment awareness	Yes	(36) 4 %	<0.0001*
	No	(14) 5.4 %	

* Statistically significant for *P* ≤ 0.05, Chi-square test, degree of freedom = 1

## Discussion

Our study provided overview about lifetime prevalence and attitudes of prohibited substances use (doping) among Saudi sport players in various sport types and from all regions of the Saudi Arabia. Our study identified that the lifetime prevalence of prohibited substances use (doping) among Saudi sport players to be 4.3 %. Predictors of prohibited substances use in our study were below university education level, age less than 20 years, the previous use any type of supplements and lack of punishment awareness. The most common reason stated for using prohibited substances was to increase performance.

It is important to estimate the prevalence and the magnitude of the doping problem in Saudi Arabia. There is a gap in knowledge to address doping violations as similar data were not reported previously from our region. There is only one pilot study addressing the use of dietary supplements but not prohibited substances in 105 football players in Riyadh; the results showed that 93.3 % athletes used different dietary supplements throughout the season, 43 % athletes reported using supplements for performance, and 32 % athletes believed in health benefits as a reason for using dietary supplements [5]. On the contrary, prevalence, knowledge and attitude of prohibited substance use among sports players have been studied previously in different countries. A study in Italy showed that 10 % of athletes admitted to frequently using amphetamines or anabolic steroids. 7 % were mentioned to be blood doping and 2 % to using beta-blockers or other classes of drugs [6]. Moreover, another French study by Laure P. estimated that the prevalence of prohibited substance use to be 3 to 5 % in children and adolescents who practicing sports [7]. This prevalence was significantly higher in boys and specifically higher in those who are in competition. Prevalence of illicit drug use in adults was found to be even higher with 5 to 15 % of adults using illegal substances. The most common drugs used were found to be stimulants, narcotics, and anabolic steroids. A cohort of 1041 professional soccer players from the two Italian major leagues was assembled during the season 2003, 92.6 % of players reported use of oral anti‐inflammatory products in the previous year with 86.1 % of them being current users. 36 % of the current users reported the use of analgesics. 82.8 % of the players reported current use of supplements, and 28 % reported using vitamins [8]. In another study from Italy; data obtained from Anti-Doping analyses performed on 100,000 urine samples from 2000 to 2009 showed that the frequency of positive finding was 1.0 to 1.8 % [9]. In a survey done in France among 1459 athletes, 4 % athletes stated that they had used doping agents at least once in their life [10]. Another study used analytical chemistry to determine the prevalence of drug abuse among elite sport students; the prevalence of positive urine samples was 11 %, and the most frequently detected compounds were the major metabolites of tetrahydrocannabinol (9.8 %) and various stimulants related to amphetamine and cocaine (1.0 %) [11]. Despite these reports, the true prevalence of doping worldwide in elite sports is unknown. A combination of questionnaires and models of biological parameters suggest that the current prevalence of intentional doping in elite athletes is 14 to 39 %. This range varies with subgroups based on type of sport. The estimated doping control test results suggest a frequency of 1 to 2 % annually [12].

The association between use of prohibited substances and lack of punishment awareness in light of the low level of advice and perception of Saudi sports players of the positive effects of substances on improving performance highlights the need for urgent awareness and educational programs on prohibited substances among sports players in Saudi Arabia. Education should be focused on the serious health consequences of using prohibited substances while awareness should be driven towards deviating perception of use of prohibited substances into taboo. Target population for awareness should focus on of younger age (less than 20 years) and those of lower education level.

Our study is the first of its kind in the region which included all types of sports not limited to one particular type of sport with large number of participants from different regions and different clubs in the kingdom of Saudi Arabia, it has increased the available data in this field, provided general insights, and identified trends about doping violations in our region. It has the limitation of being a cross sectional questionnaire based survey with possible bias of reporting because using prohibited substances is a sensitive and confidential issue which may affects the future of the players, this may led to underreporting and subsequently underestimation of the true doping prevalence in Saudi Arabia.

This study puts forward several recommendations about the need to fill gaps in knowledge, particularly from a Saudi Arabian context, and to increase the awareness of the relationship between sport participation and youth substance use. Such recommendations include increasing understanding and awareness among key stakeholders involved in youth sport of the relationship between sport participation and substance use. Moreover, SAADC should use its authorities to launch more educational programs and to study the effect of awareness campaigns on the prevalence of substance use specifically among those below 20 years of age and of low educational level. Increasing awareness and understanding about the relationship between sport participation and substance use is important to ensuring a positive sport experience for youth that is free of doping.

## Conclusion

The present study is the first study addressing the doping problem in Saudi Arabia. It confirmed that doping is a common problem in Saudi Arabia, though the prevalence is 4.3 %, it probably even higher prevalence rate considering possible underreporting of some players due to the nature of such study. Further developments in Anti-Doping screening, education and awareness are needed to ensure a safe and fair sport environment for Saudi Arabian athletes.
